# In Vitro Bioaccessibility Assessment of Phenolic Compounds from Encapsulated Grape Pomace Extract by Ionic Gelation

**DOI:** 10.3390/molecules28135285

**Published:** 2023-07-07

**Authors:** Josipa Martinović, Jasmina Lukinac, Marko Jukić, Rita Ambrus, Mirela Planinić, Gordana Šelo, Ana-Marija Klarić, Gabriela Perković, Ana Bucić-Kojić

**Affiliations:** 1 Faculty of Food Technology Osijek, Josip Juraj Strossmayer University of Osijek, F. Kuhača 18, HR-31 000 Osijek, Croatiagselo@ptfos.hr (G.Š.);; 2 Faculty of Pharmacy, Institute of Pharmaceutical Technology and Regulatory Affairs, University of Szeged, H-6720 Szeged, Hungary

**Keywords:** grape pomace extract, phenolic compounds, encapsulation, ionic gelation, natural coatings, bioaccessibility, in vitro simulated digestion

## Abstract

Grape pomace is a by-product of winemaking characterized by a rich chemical composition from which phenolics stand out. Phenolics are health-promoting agents, and their beneficial effects depend on their bioaccessibility, which is influenced by gastrointestinal digestion. The effect of encapsulating phenol-rich grape pomace extract (PRE) with sodium alginate (SA), a mixture of SA with gelatin (SA-GEL), and SA with chitosan (SA-CHIT) on the bioaccessibility index (*BI*) of phenolics during simulated digestion in vitro was studied. A total of 27 individual phenolic compounds (IPCs) were quantified by UHPLC. The addition of a second coating to SA improved the encapsulation efficiency (*EE*), and the highest *EE* was obtained for SA-CHIT microbeads (56.25%). Encapsulation affected the physicochemical properties (size, shape and texture, morphology, crystallinity) of the produced microbeads, which influenced the delivery of phenolics to the intestine and their *BI*. Thus, SA-GEL microbeads had the largest size parameters, as confirmed by scanning electron microscopy (SEM), and the highest *BI* for total phenolic compounds and IPCs (gallic acid, 3,4-dihydroxybenzoic acid and *o*-coumaric acid, epicatechin, and gallocatechin gallate) ranged from 96.20 to 1011.3%. The results suggest that encapsulated PRE has great potential to be used as a functional ingredient in products for oral administration.

## 1. Introduction

Grape pomace is a by-product of winemaking that has attracted much attention in the last three decades due to its chemical composition and great potential for reuse in the production of high-value products (enzymes, dietary supplements, biofuels, biopolymers, etc.). It consists of the skins, seeds, pulp, and sometimes the stems of the grapes, which influence its chemical composition, rich in many different components such as sugars, fibers, fats, proteins, bioactive phenolic compounds, etc. [1,2,3]. Scientific interest in phenolic compounds from grape pomace is most prominent in the literature and has not diminished over the years, as they offer numerous benefits when used in the pharmaceutical, food, and cosmetic industries, or in medicinal applications. It is estimated that 60–70% of the phenolic compounds present in grapes remain in the pomace after wine production [3,4]. Phenolic compounds are known for their multiple health-promoting effects (antioxidant, anti-cancer, anti-inflammatory, antiviral, etc.), but their low bioaccessibility in the human gastrointestinal system limits their beneficial effects [5]. The bioaccessibility of a bioactive compound refers to the proportion of the compound that is released from a matrix and becomes available for absorption during digestion in the gastrointestinal tract and transport through various tissues of the human body [5]. During this process, many changes occur, such as changes in temperature, pH, ionic strength, and other metabolic factors that affect the stability of phenolic compounds. Additionally, the phenolic compounds usually interact with other components of the matrix in which they are found, and the presence of enzymes (e.g., pepsin, pancreatin) and bile salts also affect these interactions [6,7]. The bioaccessibility of phenolic compounds can be compromised by several factors, including the matrix in which they are found and the physicochemical properties of the phenolic compounds (durability under gastrointestinal conditions, rapid metabolism, low water solubility, poor absorption). These challenges can be overcome through encapsulation of phenolic compounds, and one promising encapsulation technique is ionic gelation [8].

Encapsulation involves incorporating a bioactive ingredient into a network with a polymer, creating a matrix that allows the bioactive ingredient to remain stable under various conditions to which it may be exposed during digestion. The tendency today is to use polymers (coatings) derived from natural sources such as sodium alginate (SA) obtained from brown algae, gelatin (GEL) from cold fish skin, and chitosan (CHIT). SA is a polysaccharide that has numerous industrial applications due to its gelling, stabilizing, and viscosity-increasing properties and its ability to bind water [9]. It is widely used for encapsulation of active compounds and is very suitable as an immobilization matrix for numerous drug delivery applications [10], cell, tissue, or gene engineering [11], site-specific delivery [12], and others. CHIT, also a polysaccharide, is derived from deacetylated chitin [13]. Due to its easy degradability, low toxicity, good biocompatibility, and low price [14], it is widely used for encapsulation to produce hydrogels used for targeted and controlled release of drugs [15], enzyme immobilization [16], tissue engineering [17], and other purposes. Unlike SA and CHIT, GEL is a protein by structure. Recently, the production of GEL from fish waste has shown great potential because it helps reduce environmental pollution, there is less risk of disease transmission compared to the use of bovine bone gelatin, there are no religious barriers, and it is widely available [18,19]. The coating used has a great impact on the protection, retention and release of the encapsulated bioactive compound by influencing the physical and chemical properties of the particles produced [20]. The dimensions of the particles also have a great influence on the release of bioactive substances; for example, increasing the size of the particles increases the possibility of a slower release of the active substances [21]. It is necessary to know the effects of different coatings on the physicochemical properties of the particles to be produced in order to control their properties and ensure the desired characteristics with regard to the further use of the particles [20].

In this work, the encapsulation of phenol-rich grape pomace extract (PRE) powder was performed by the ionic gelation method using SA, a combination of SA with GEL, and SA with CHIT, followed by simulated digestion in vitro to study the effect of encapsulation on the bioaccessibility of the phenolic compounds compared to a PRE. The physicochemical properties of the microbeads produced were also analyzed. As far as we know, there is little literature dealing with the encapsulation of PRE using the ionic gelation method, and this study contributes to the body of knowledge on improving the bioaccessibility of phenolic compounds encapsulated under the conditions studied.

## 2. Results and Discussion

### 2.1. Chemical Composition of Grape Pomace

The chemical composition of grape pomace is shown in Table 1. The crude protein, free fat, and ash contents in grape pomace were 8.72%_db_, 8.54%_db_, and 7.16%_db_, respectively. Of the 13 total sugars studied, four were quantified, and it can be seen that sucrose was found in the highest concentration (9.87 mg/g_db_) and arabinose in the lowest concentration (1.50 mg/g_db_). The results obtained are in agreement with the studies of other authors, with differences related to sample variety, geographical and climatic factors, cultivation, and vinification processes. For example, Jin et al. [22] studied different varieties of white and black grape pomace from Virginia (USA), including the Cabernet Franc variety. When examining chemical composition, they obtained very similar results in terms of crude protein (13.0%_db_) and ash (5.24%_db_), while only three sugars were determined: sucrose (1.70 mg/g_db_), glucose (2.31 mg/g_db_), and fructose (3.79 mg/g_db_).

Due to the presence of these nutritive components, grape pomace can be used in the production of animal feed or other value-added food products (e.g., functional food, dietary supplements, etc.) [23,24,25,26,27] as an effective way to reduce feed or food costs. In addition, the total nitrogen content (0.96 mg/g_db_) and carbon content (53.32–59.22 mg/g_db_) determined in this study indicate the potential of using grape pomace as a biofertilizer [28] contributing to the zero-waste concept and enriching soil composition.

Grape pomace is often referred to as lignocellulosic material because it contains many insoluble fibers—hemicellulose, cellulose, and lignin—which is also confirmed by their percentage in the tested sample (Table 1): 9.64%_db_, 13.82%_db_, and 23.77%_db_, respectively. It has already been proven that the grape pomace extract, rich in these fibers, has high antioxidant activity due to the phenolic acids contained in the lignin structure [29], and for this very reason, has a positive effect on human health. They are believed to play an important role in the gastrointestinal system by modulating transit time, affecting faecal acidity and intestinal microflora [30]. Sánchez-Tena et al. [31] found a strong antitumor activity of freeze-dried grape pomace related to the synergistic action of the fibers and the phenolic compounds contained in these fibers. Furthermore, Martín-Carrón et al. [32] demonstrated in normo- and hypercholesterolemic rats that the fibers contained in grape pomace lower cholesterol levels. In addition to these effects, the insoluble fibers of grape pomace can also be used for other applications, e.g., they can be isolated and used again as a clarifying agent for red wines due to their ability to adsorb tannins [33]. In this study, phenolic compounds are considered as the most valuable components of grape pomace, and the results are given Section 2.4.

### 2.2. Encapsulation Efficiency of Grape Pomace Extract

**Figure 1:**
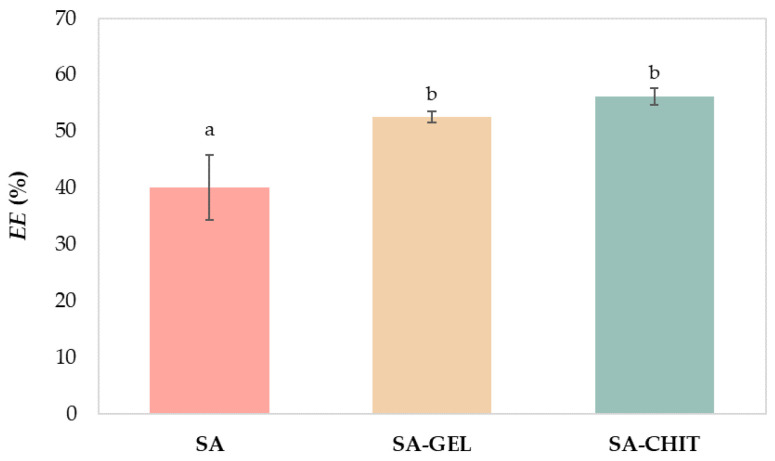
Encapsulation efficiency (*EE*, %) of phenol-rich grape pomace extracts using 3% (*w/v*) sodium alginate (SA) and its blends with 5% (*w/v*) gelatin (SA-GEL) and 1.5% (*w/v*) chitosan (SA-CHIT). Different letters (a, b) represent statistically significant differences between results (ANOVA, post-hoc Duncan test at *p* < 0.05).

SA and its mixtures, SA-GEL and SA-CHIT, were used to perform the ionic gelation method with the aim of encapsulating PRE. To determine the percentage of phenolic compounds found in the encapsulate, after the preparation of hydrogels, the encapsulation efficiency (*EE*) was calculated. The results showed that it is possible to increase the *EE* by adding an additional polymer to SA (Figure 1).

When SA was used as the sole coating, there was an *EE* of 40.08%, which was statistically significantly different (Duncan test at *p* < 0.05) from the *EE* when GEL and CHIT were combined with SA. Higher *EE* was obtained by SA-GEL (52.63%) and SA-CHIT (56.25%), and they were not statistically significantly different from each other. The results obtained are comparable to many studies in the literature that have shown that the use of several different coatings can result in the higher *EE* of an active ingredient. Balanč et al. [34] encapsulated Carqueja extract by the ionic gelation method using SA and obtained an *EE* of 49%, while the *EE* was 73.8% when inulin was added as an additional coating. Navarro-Flores et al. [35] encapsulated phenolic compounds from the leaves of *Crotalaria longirostrata* using the spray drying method and obtained an *EE* of 92% using soy protein with maltodextrin, while Pashazadeh et al. [36] used only maltodextrin for the encapsulation of phenolic compounds from corn husks and obtained an *EE* of 88.29%.

Furthermore, the solubility of the coating used and the active ingredient, as well as their interactions, are very important for the *EE* [37]. For example, it is known that phenolic compounds and proteins can link by hydrogen and hydrophobic bonds, and proteins can also be bound to free carboxyl groups of other coatings such as SA. This was confirmed by a study conducted by Belščak-Cvitanović et al. [38], which investigated how the use of different proteins with SA affects the retention of phenolic compounds from green tea. The results showed that the *EE* was 66.1% when SA was used, and with the addition of whey proteins or calcium caseinate, the *EE* increased to 76.5% and 77.2%, respectively. Another example is interactions that occur between SA and CHIT. Studies have shown that chitosan can form a membrane on the prepared alginate hydrogels due to its cationic nature and protonated amino groups by forming a complex with free carboxyl groups of anionic alginate [39]. Although many studies show an increase in *EE* when chitosan is used, a study conducted by Deladino et al. [40] found a lower *EE* when chitosan was used to encapsulate yerba mate polyphenols. Such discrepancies in the results obtained are common, possibly due to the use of different concentrations of coatings, encapsulation techniques, and process parameters.

### 2.3. Physicochemical Characterization of Microbeads

#### 2.3.1. Geometric Parameters, Texture, and Morphology of Microbeads

Geometric parameters (size and shape) of produced microbeads are physical properties that depend on the encapsulation method and the coating used. These parameters have great influence on the release of the active ingredient—in this case, phenolic compounds. For example, produced microbeads with a higher *EE* often have a greater thickness, which then causes a slower release, while beads with a smaller size have a larger contact area, which can lead to a faster release of the active ingredient [41]. According to the size parameters of the microbeads, SA-GEL microbeads showed the highest values of all measured parameters (area, perimeter, and Feret), whereas SA microbeads showed the lowest values (Table 2). Statistical analysis shows that there is a statistically significant difference between size parameters of microbeads produced with different coatings, except for Feret_MAX_, where there is no statistically significant difference between SA and SA-CHIT microbeads.

In addition, when the shape parameters were examined, it was found that the SA microbeads had the largest values for circularity, which is a measure of the deviation of the particles from spherical, indicating that the SA microbeads had the most spherical shape compared with SA-GEL and SA-CHIT, which were not statistically different (Duncan test at *p* < 0.05) (Table 2). The geometry of microbeads is a simple physical phenomenon that affects the pharmacodynamic and pharmacokinetic properties of the encapsulated material. Changes in geometry can affect the interactions of microbeads with cells or proteins and, in general, biodistribution in the gastrointestinal system [42].

**Figure 2:**
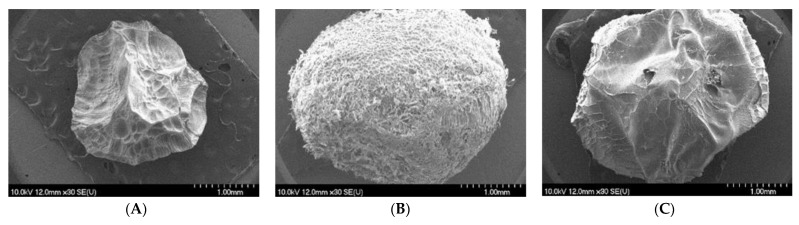
SEM images of microbeads with phenol-rich grape pomace extract encapsulated with sodium alginate (**A**); sodium alginate with gelatin (**B**) and with chitosan (**C**); and their outer layer with a scale of 1 mm and 50 µm for each sample.

According to the determined values for shape parameter roundness, which represents the curvature of the particle edges, the SA-GEL and SA-CHIT microbeads are almost indistinguishable, while in comparison with the SA microbeads, there is a statistically significant difference (Duncan test at *p* < 0.05). The solidity parameter is a measure of the smoothness of the outline of particles studied, and in this case, there is a significant difference between all samples, with SA-GEL microbeads having the smoothest outline. The images obtained with Scanning Electron Microscopy (SEM) confirm the data obtained for the size parameters of the microbeads, i.e., it can be seen that the SA microbeads are smaller than the other microbeads (Figure 2).

It is also noted that SA-GEL microbeads have a more wrinkled surface but a more spherical shape, while SA and SA-CHIT microbeads have depressions on the surface. It can be noticed that the SA-CHIT microbeads have what appears to be the smoothest surface compared to the other microbeads. In addition to the parameters of size and shape, the surface of microbeads also plays a role in the release. Anything that causes the wrinkled surface of the microbeads, as seen on SA and SA-GEL, also causes an additional increase in contact area during in vitro release, allowing for faster release of the encapsulated component.

When texture was determined, the results showed that SA microbeads had the highest hardness (1.36 N), while the hardnesses of SA-GEL and SA-CHIT microbeads were 63.97% and 58.82% lower, respectively (Table 2). The hardness of the SA microbeads was statistically significantly different from those of the SA-GEL and SA-CHIT microbeads. It is known that a harder matrix tends to hold the encapsulated ingredients more tightly, i.e., release them slowly, but this was not the case in this work (as described in 2.5) due to the influence of many other factors described previously (size, shape, and morphology of microbeads).

#### 2.3.2. X-ray Powder Diffraction and Differential Scanning Calorimetry Analyses

**Figure 3:**
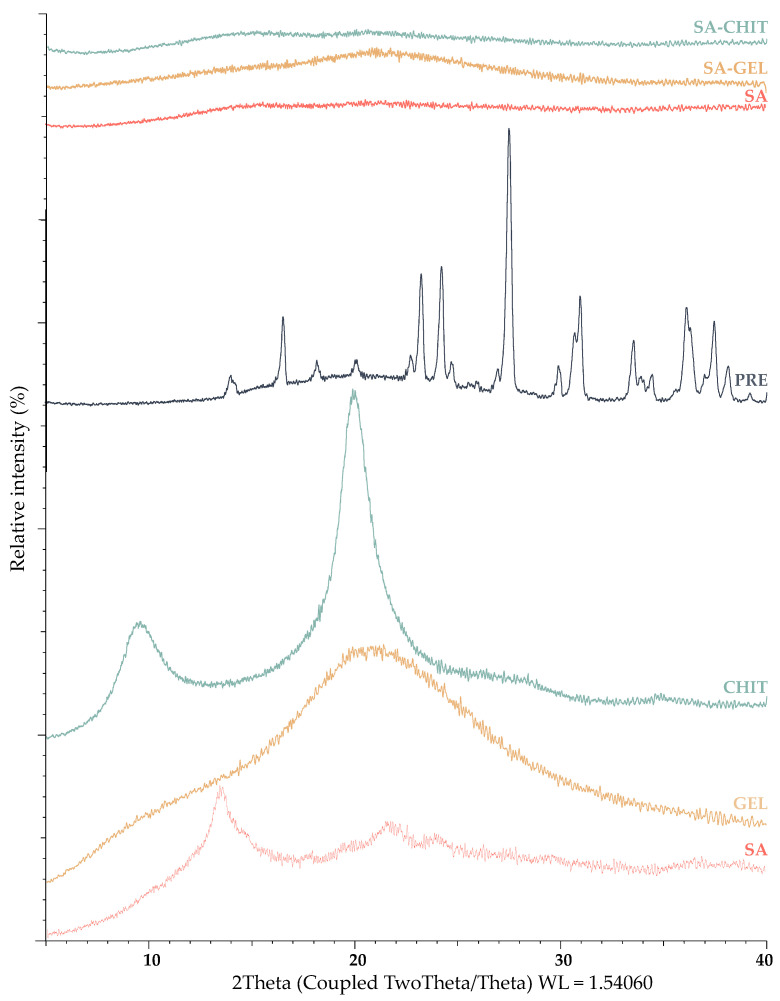
X-ray powder diffractograms of phenol-rich grape pomace extracts (PRE), pure coatings (sodium alginate, SA; gelatin, GEL; and chitosan, CHIT), and microbeads containing PRE prepared using various coatings (SA, SA-GEL, and SA-CHIT).

X-Ray Powder Diffraction (XRPD) and Differential Scanning Calorimetry (DSC) analyses were used to investigate the crystallinity and amorphousness of the PRE and the coatings and produced microbeads containing PRE. The results of the XRPD analysis show that coatings of sodium alginate and gelatin have an amorphous structure, and chitosan has a semicrystalline structure, while sharp peaks indicate a crystalline structure of the PRE (Figure 3).

**Figure 4:**
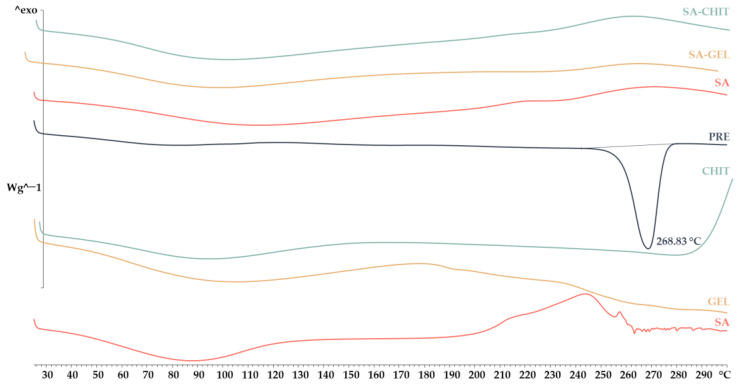
Differential scanning calorimetry thermograms of phenol-rich grape pomace extract (PRE), pure coatings (sodium alginate, SA; gelatin, GEL; and chitosan, CHIT), and microbeads containing PRE prepared using various coatings (SA, SA-GEL, and SA-CHIT).

The study of the structure of all microbeads containing PRE shows the stable amorphous form, as the structure did not change after 3 months. These results were also confirmed by DSC analysis (Figure 4). Also evident from the DSC curves are slightly broad peaks for the coating and microbeads, indicating water loss, and for the extract, an endothermic peak is visible with a transition at 268.63 °C, indicating melting of the PRE. Many studies have confirmed these changes from crystalline to amorphous structure during encapsulation [43,44]. Indeed, this change plays an important role in the digestibility of the PRE, since the extract in crystalline form is less available for absorption due to the reduced possibility of passage through the intestinal membrane.

### 2.4. Phenolic Content of Grape Pomace Extract before In Vitro Digestion

#### 2.4.1. Total Phenolic Compounds

**Figure 5:**
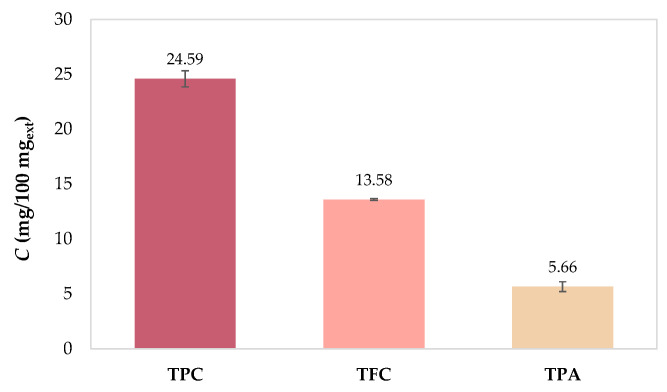
Total phenolic compound content (TPC); total flavonoid content (TFC) and total extractable proanthocyandin content (TPA) of phenol-rich grape pomace extract before in vitro digestion.

The total phenolic content (TPC), total flavonoid content (TFC), and total extractable proanthocyanidin content (TPA) of PRE were determined spectrophotometrically (Figure 5).

The results showed that TPC (24.59 mg_GAE_/100 mg_ext_) is the most abundant, followed by TFC (13.58 mg_CE_/100 mg_ext_) and TPA (5.66 mg/100 mg_ext_). The results available in the literature vary widely because they depend on many factors, including cultivar and climatic conditions, agrotechnical conditions, geographical location, winemaking process, extraction procedure, etc. For example, Xu et al. [45] also studied the composition of Cabernet Franc from Orange County (Orange, VA, USA) and found TPC 15.38 mg_GAE_/100 mg_ext_ and TFC 9.17 mg_CE_/100 mg_ext_, while Jin et al. [22] obtained 36.1 mg_GAE_/g_db_ for TPC, 16.3 mg_CE_/g_db_ for TFC, and 21.2 mg/g_db_ for TPA with Cabernet Franc from Crozet (Crozet, VA, USA). The content and profile of individual phenolic compounds (IPCs) of PRE were determined by ultra-high performance liquid chromatography (UHPLC), and the results are presented in Table 3. Of the 33 phenolic compounds tested, 27 compounds were quantified and divided into two main groups: non-flavonoids (phenolic acids and stilbenes) and flavonoids (flavan-3-ols, flavonols, anthocyanins) (Table 3).

#### 2.4.2. Individual Phenolic Compounds

The major non-flavonoid subgroup of phenolic compounds is the phenolic acids, which are divided into hydroxycinnamic acids and hydroxybenzoic acids. A total of 10 phenolic acids, namely six hydroxybenzoic acids (gallic acid, 3,4-dihydroxybenzoic acid, *p*-hydroxybenzoic acid, syringic acid, vanillic acid, and ellagic acid) and four hydroxycinnamic acids (caffeic acid, ferulic acid, and *o*- and *p*-coumaric acids) were found from 13 acids tested. Many of these phenolic acids are used in various applications, such as skin care products or as food preservatives, but due to their multiple contributions to human health through their anticancer, antioxidant, antimicrobial, neuroprotective, anti-inflammatory, and antidiabetic activities [46,47], they can also be used in medical or pharmaceutical treatments. In general, the content of hydroxybenzoic acids in PRE was dominant compared with hydroxycinnamic acids (Table 3). Gallic acid was found in the highest concentration (130.00 μg/100 mg_ext_), followed by syringic and ellagic acid, with concentrations of 114.36 μg/100 mg_ext_ and 81.33 μg/100 mg_ext_, respectively. Gallic acid is naturally present in many fruits and vegetables and has numerous biological functions, including anticancer, antimicrobial, and antioxidant effects, to name a few. Together with its derivatives, gallic acid has been shown to have beneficial effects on immune response and the gut microbiome, but its poor solubility, stability, and bioavailability, as well as its rapid metabolism and excretion, present barriers to exploiting these properties [48]. Syringic acid is known for alleviating oxidative stress [49,50], hepatoprotective [51], antidiabetic [52], anti-inflammatory [53], neuroprotective [54], and antiendotoxic [55] activity, but it is characterized by lipophilicity and rapid excretion, resulting in low bioavailability, and thus low therapeutic effect [56]. Ellagic acid is a smaller molecule than the ellagitannin from which it can be obtained, but it has much lower solubility, and thus bioavailability. It cannot be absorbed in the stomach or small intestine, and therefore enters the colon, where it undergoes numerous metabolic changes by the intestinal microbiota, and urolithins are formed. Despite similar health properties to ellagic acid, urolithins are not recommended for therapeutic use and may be potentially harmful. For this reason, many studies have focused on producing formulations that allow ellagic acid to be released at the desired site in the gastrointestinal tract [57]. 3,4-Dihydroxybenzoic acid and vanillic acid were found with contents of 24.09 μg/100 mg_ext_ and 14.83 μg/100 mg_ext_, respectively, both of which have anti-inflammatory, neuroprotective, antidiabetic, and antioxidant properties [58,59]. The highest content from the hydroxycinnamic phenolic acid group was found for *o*-coumaric acid, with a content of 19.48 μg/100 mg_ext_, followed by *p*-coumaric acid, ferulic acid, and caffeic acid, with the lowest content (1.04 μg/100 mg_ext_) (Table 3). Although they are not found in very high concentrations in grape pomace, hydroxycinnamic acids are extremely important, as they have many beneficial biological activities such as antioxidant and antitumor properties, and also contribute to the prevention of cardiovascular diseases and high blood pressure [60,61,62]. In addition, a diet rich in hydroxycinnamic acids reduces the risk of Alzheimer’s disease and atherosclerosis [63,64].

Stilbenes are the second subgroup of non-flavonoids found in grape pomace, with resveratrol being the most studied. Like other phenolic compounds, stilbenes exhibit great antioxidant activity, but also contribute to the prevention of cancer and cardiovascular diseases and have neuroprotective and anti-inflammatory properties [65]. In this study, resveratrol and its dimer, ε-viniferin, were also observed, with ε-viniferin found at higher concentrations (13.72 μg/100 mg_ext_) (Table 3).

Of 17 flavonoids tested, 15 were identified: flavan-3-ols (epicatechin, catechin, epicatechin gallate, galloctechin gallate, and procyanidins B1 and B2), anthocyanins (oenin chloride, myrtillin chloride, kuromanin chloride, petunidin chloride, callistephin chloride, and peonidin-3-*O*-glucoside chloride), and flavonols (quercetin, kaempferol, and rutin).

Flavan-3-ols present in grape pomace are mainly in the form of catechins and account for 13–30% of the total phenolic content in red grapes, while their content is higher in white grapes (46–56%) [66], and in this study, epicatechin (547.27 μg/100 mg_ext_) and catechin (527.59 μg/100 mg_ext_) were the most abundant phenolic compounds, followed by procyanidin B1 (317.42 μg/100 mg_ext_), gallocatechin gallate (127.23 μg/100 mg_ext_), procyanidin B2 (126.51 μg/100 mg_ext_), and epicatechin gallate (Table 3). Heptinstall et al. [67] showed that flavanols are of great interest by demonstrating in vitro and ex vivo that cocoa flavanols can affect platelet and leukocyte function and thus improve cardiovascular health. In addition, proanthocyanidins show strong anticancer, antimicrobial, and chemoprotective activities, as well as a strong antioxidant effect [55,68,69,70].

The flavonols quantified in PRE are quercetin, rutin, and kaempferol, with quercetin found in the highest concentrations (146.04 μg/g_db_), as seen from Table 3. The anti-osteoporotic activity of these compounds has been demonstrated in in vitro and in vivo studies [71].

A further subgroup of flavonoids is the anthocyanins, which are usually associated with the color of red grapes and can be used as natural pigments. However, they also contribute to health with their cardioprotective, antithrombotic, antiatherosclerotic, vasoprotective, antilipemic, anti-inflammatory, and other properties [72]. Large variances in content of anthocyanins in PRE were found (Table 3). Oenin chloride was the dominant anthocyanin in PRE, with a content of 794.37 μg/100 mg_ext_, while the concentration of the least represented anthocyanin, callistephin chloride, was 2.27 μg/100 mg_ext_. Oenin chloride is an anthocyanin present in the highest concentrations in most red grape varieties, while other compounds vary by grape variety, but myrtillin chloride and peonidin-3-*O*-glucoside are commonly found in the composition of grape pomace. Fabroni et al. [73] found an association between an anthocyanin-rich extract and an anti-obesity effect through inhibition of pancreatic lipase activity. Flavonols and flavan-3-ols have been attributed similar properties to anthocyanins, e.g., antioxidant, antiviral, antithrombotic, anticancer, and cardio-protective properties [74,75,76,77].

### 2.5. In Vitro Simulated Digestion and Bioaccessibility Index of Phenolic Compounds

**Figure 6:**
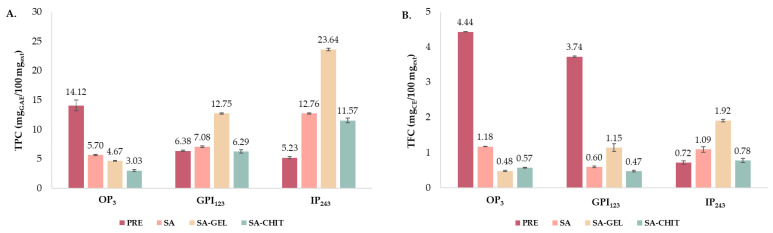

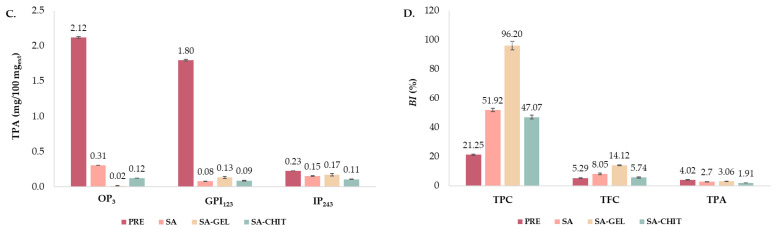
Total phenolic compound content (TPC)—(**A**); total flavonoid content (TFC)—(**B**); and total extractable proanthocyandin content (TPA)—(**C**) at the end of oral (OP_3_), gastric (GP_123_) and intestinal phase (IP_243_) of in vitro simulated digestion of phenol-rich grape pomace extract (PRE) and microbeads containing PRE prepared with sodium alginate (SA), SA with gelatin (SA-GEL), and SA with chitosan (SA-CHIT), and bioaccessibility index (*BI*) after IP—(**D**) for TPC, TFC, and TPA.

**Figure 7:**
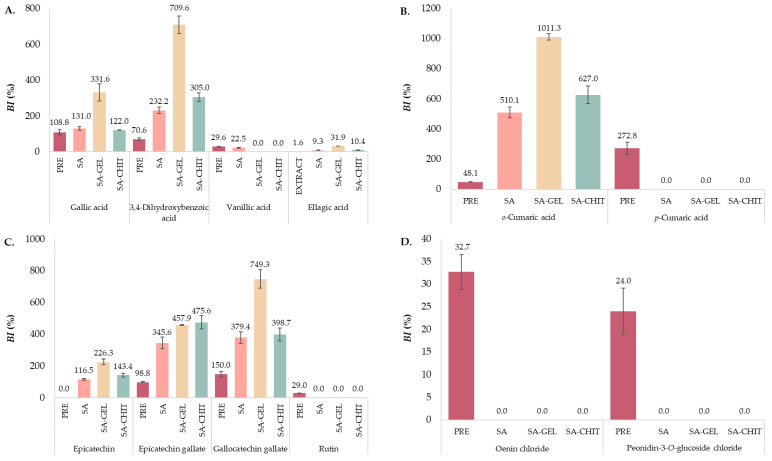
Bioaccessibility index (*BI*, %) of hydroxybenzoic acids (gallic, 3,4-dihydroxybenzoic, vanillic and ellagic acid)—(**A**); hydroxycinnamic phenolic acids (*o*- and *p*-coumaric acids)—(**B**); flavan-3-ols (epicatechin, epicatechin gallate, gallocatechin gallate) and flavonol rutin—(**C**); and anthocyanins (oenin chloride and peonidin-3-*O*-glucoside chloride)—(**D**) after in vitro simulated digestion of phenol-rich grape pomace extract (PRE) and microbeads containing PRE prepared with sodium alginate (SA), SA with gelatin (SA-GEL), and SA with chitosan (SA-CHIT).

Simulated digestion in vitro is a widely used method to estimate the bioaccessibility of target compounds. It can provide more information about the metabolism of phenolic compounds, their availability for further absorption in the body, and the possibility of having beneficial effects on human health. To become bioaccessible, phenolic compounds must be released from the matrix during gastrointestinal digestion. Therefore, in this study, the behavior of PRE and microbeads (SA, SA-GEL, and SA-CHIT) containing PRE was investigated during simulated digestion in vitro. Samples were collected for 243 min, which included oral (OP) which lasted 3 min; gastric phase (GP), 120 min; and intestinal phase (IP), 120 min, to evaluate the content of TPC, TFC, TPA (Figure 6), and IPCs (Table 3), while the bioaccessibility index (*BI*) was also calculated (Figure 6D and Figure 7).

#### 2.5.1. Total Phenolic Compounds

In vitro simulated digestion of PRE showed a pattern of decreasing TPC (Figure 6A), although the TPC of all microbeads continuously increased, with SA-GEL having the greatest content at the end of digestion (IP_243_, 23.64 mg_TPC_/100 mg_ext_). Figure 6B,C show that TFC and TPA of PRE decreased during the digestion; however, the case for microbeads was different. After SA microbead digestion, TFC and TPA in GP_123_ decreased, followed by an increase in IP_243_. TPA content increased throughout the digestion of SA-GEL microbeads but not of SA-CHIT microbeads. Figure 6D indicates that compared to the PRE, the use of SA-CHIT, SA, and SA-GEL enhances the *BI* of TPC by 2.2, 2.4, and 4.5 times, respectively. There was a 2.7-fold increase in *BI* for TFC with SA-GEL microbeads, but no increase in *BI* for TPA with encapsulated extract. Several studies are available in the literature in which a phenol-rich extract has been successfully encapsulated and the *BI* of TPC has been increased. Thus, Aguilera-Chávez et al. [78] encapsulated phenol-rich extract from the Mexican plum ecotype “Cuernavaqueña” using gum arabic as a coating in spray-drying and spout-fluid-bed drying and obtained a *BI* for TPC of 61.35% and 63.61%, respectively.

In addition, phenolic compounds extracted from cocoa hull waste were encapsulated in liposomes. The prepared liposomes were then coated with chitosan using a layer-by-layer method and spray-dried with maltodextrin to investigate how these methods affect the bioaccessibility of phenolic compounds. It was found that encapsulation increased the *BI* of TPC and TFC from cocoa hull waste by 6- and 8-fold, respectively [79]. There is, however, a lack of literature and data regarding grape pomace encapsulated by ionic gelation, and the findings presented in this work can be considered a novel contribution to this field.

#### 2.5.2. Individual Phenolic Compounds

##### Phenolic Acids

Of the six hydroxybenzoic phenolic acids found in the PRE before digestion, five were found during digestion of PRE and microbeads containing PRE. Gallic acid, the most abundant phenolic acid in the extract, was released at low concentrations during OP and GP, while a high release was observed during IP (Table 3). The increase in gallic acid content during IP could be due to the degradation of anthocyanins such as oenin chloride and myrtillin chloride [80,81]. The greatest increase in the content of gallic acid was achieved by encapsulation with SA-GEL, with a 3-fold increase in *BI* compared to PRE (Figure 7A). A similar case to gallic acid was observed with 3,4-dihydroxybenzoic acid, which was not detected in any sample until IP, and then it was detected in much higher concentrations (1.67–5.37 times higher) than in the PRE before digestion.

This phenolic acid is also a degradation product of oenin chloride, but also of kuromanin chloride [81], which was detected only in the PRE before digestion (Table 3). All this resulted in a high *BI* of 3,4-dihydroxybenzoic acid, with the highest increase of more than 10-fold (compared to the PRE) when SA-GEL was used to encapsulate the PRE (Figure 7A). Furthermore, syringic acid was released in OP and GP from all microbeads but not in IP, while on the other hand, vanillic acid was detected only in IP released from SA microbeads and in IP_243_ released from PRE (Table 3). During OP, ellagic acid was observed only from SA microbeads (1.72 μg/100 mg_ext_), and throughout the rest of the in vitro digestion, it was detected in all samples except GP_33_ for SA-GEL microbeads and GP_123_ for PRE. The release of this phenolic acid was highest at the beginning of IP (IP_153_) from all microbeads, followed by a decrease. Despite the decrease during IP, in terms of the *BI* of ellagic acid after digestion of the PRE, encapsulation improved the *BI* of ellagic acid by 5.8-fold when SA was used as a coating, by 6.5-fold when SA-CHIT was used, and even by 19.9-fold when SA-GEL was used (Figure 7A).

Of the four hydroxycinnamic phenolic acids found in the PRE (Table 3), only *o*-coumaric acid was detected during digestion of the microbeads. Already in GP_63_ with SA-CHIT microbeads, it was released at higher content (21.11 μg/100 mg_ext_) than found in the PRE before digestion. In the IP, the content of *o*-coumaric acid increased significantly, and the highest content was released from SA-CHIT microbeads (123.03 μg/100 mg_ext_). From the PRE, *o*-coumaric acid was released only at the end of IP, while in OP and GP it was not detected. Moreover, *p*-coumaric acid was detected in all three phases only during digestion of the PRE, with a 9.1-fold increase in content at the end of IP compared to OP and GP, but also a 2.7-fold increase compared with the PRE before digestion. Figure 7B shows a large difference between the *BI* values of *o*- and *p*-coumaric acids from the PRE. While the encapsulation had no effect on the *BI* of *p*-coumaric acid, encapsulation successfully improved the *BI* of *o*-coumaric acid, with the highest obtained with SA-GEL coatings (1011.3%). *BI* values above 100%, as determined for individual phenolic acids (gallic acid, 3,4-dihydroxybenzoic acid, *o*- and *p*-coumaric acid), indicate that more complex phenolic compounds were degraded during simulated digestion, resulting in the release of simpler compounds.

##### Flavan-3-ols

The two flavan-3-ols most abundant in the PRE, epicatechin and catechin, showed different release trends during the in vitro digestion simulation (Table 3). The content of released epicatechin from the PRE in GP was 5.55% lower compared with OP and was undetectable at the end of IP, due to its instability during the transition from GP to IP and under IP conditions [82], whereas an opposite trend was observed in microbeads. After the release in OP from all microbeads, the release in GP continues, and the highest concentrations obtained were 127.29 μg/100 mg_ext_ in GP_93_ from SA-CHIT microbeads, 157.62 μg/100 mg_ext_ in GP_123_ from SA, and 133.18 μg/100 mg_ext_ in GP_123_ from SA-GEL, respectively. When IP is observed, the concentration of epicatechin continues to increase, reaching a content of 685.58 μg/100 mg_ext_, 634.47 μg/100 mg_ext_, and 790.51 μg/100 mg_ext_ released from SA, SA-GEL, and SA-CHIT microbeads, respectively. This increase in epicatechin concentration can be explained by the degradation of procyanidin B2 during GP, which is described in more detail below. For this reason, high *BI*s of epicatechin were also obtained after digestion of the encapsulated PRE (Figure 7C). In contrast to epicatechin, catechin was released in OP and during GP from the prepared microbeads and PRE but was not detected during IP. Epicatechin gallate during digestion of PRE was only detected at the very end of the IP (32.06 μg/100 mg_ext_), with nearly identical content determined in the PRE before digestion (32.46 μg/100 mg_ext_). However, upon digestion of the encapsulated PRE, there is a clear trend toward an increase in epicatechin gallate concentration by the end of GP, and a decrease in IP. The highest *BI* of epicatechin gallate was obtained after in vitro digestion of SA-CHIT microbeads (475.6%), which was more than 4.8 times higher than the *BI* observed after digestion of the unencapsulated extract. Gallocatechin gallate was found only in the IP during digestion of the PRE, SA, and SA-GEL microbeads. An exception was the SA-CHIT microbeads, where gallocatechin gallate was detected in all phases of digestion. From the results, encapsulation improved the gallocatechin gallate *BI* (Figure 7C). SA and SA-CHIT microbeads showed similar results, i.e., 2.5- and 2.7-times higher *BI*, respectively, compared with the PRE, while the *BI* for SA-GEL microbeads was almost five times higher than the value observed with the PRE. Many studies [83,84] have shown the high bioaccessibility of catechins in fruits is attributed to the hydrolysis of polymerized compounds such as procyanidins [85]. However, the structure of the plant matrix and the binding of the gallate components of flavan-3-ol to the material of the plant cell wall also have a great influence on the bioaccessibility of these compounds, according to de Lima Oliveira et al. [86]. In addition, de Lima Oliveira et al. [86] point out that the fibers form pores in their structure into which phenolic compounds can penetrate, so that the proportion of fibers in the plant material influences the bioaccessibility of flavan-3-ols. Two procyanidins, B1 and B2, were tested, both of which were found in the PRE before and during digestion in OP and GP, but mainly in GP (Table 3). Although procyanidin B1 was found at a higher content in the PRE, its content was 46.11% lower than that of procyanidin B2 when the extract was digested in OP and 47.28% lower in GP. Procyanidin B1 was observed only during OP from SA microbeads. In contrast, procyanidin B2 was detected in OP and GP during digestion of SA and SA-CHIT microbeads, and for SA-GEL microbeads from GP_63_. Moreover, it was not identified in any sample during the intestinal phase, except in IP_153_ from SA microbeads. Procyanidin B2 has been reported [82] to be degraded to (-)-epicatechin over time under conditions found in the upper part of the gastrointestinal tract and when exposed to mixing, such as during in vitro digestion simulation, which is consistent with the presented results.

##### Flavonols

The flavonols quercetin and rutin were quantified only during digestion of PRE (Table 3), whereas kaempferol was not determined during digestion. Compared with OP, by the end of GP, the content of quercetin decreased by 19.01%, while it could not be detected at the end of IP. In contrast, the content of rutin in GP increased by 37.13% compared with OP and then decreased significantly in IP (70.62% compared to GP). Both flavonols are characterized by poor permeability, low water solubility, and poor stability, which causes their low bioaccessibility [87,88], as shown by the results of this study. Rutin has been shown to have a *BI* value of 29.0% after in vitro digestion of the PRE (Figure 7C), but the selected coatings have not been shown to have a beneficial effect on increasing the *BI* of rutin.

##### Anthocyanins

Anthocyanins are water-soluble, protonated molecules that are sensitive to different pH values and therefore undergo numerous changes during digestion. It has been reported that the weak basic pH of saliva may have some effect on anthocyanins [89], although they have been reported to be relatively stable under gastric conditions [90]. Their structure consists of an anthocyanidin conjugated to a sugar moiety(s), and it has been suggested that degradation of anthocyanins in the intestinal tract begins with cleavage of this sugar moiety [91]. The aglycones formed are not stable under the neutral conditions of the intestine and are rapidly converted to phenolic acids and phenolic aldehydes by further cleavage [92]. Table 3 shows that myrtillin chloride and petunidin chloride were detected only in the PRE during OP and GP, where the content of myrtillin chloride decreased by 15.66% by the end of GP, while the content of petunidin chloride increased by 16.40% compared with OP. Moreover, no release of oenin chloride from SA-CHIT microbeads was observed in the OP, but during digestion, the highest content was released in GP_63_, which then decreased until the end of GP_123_. A similar trend was observed with SA microbeads, where the highest content of oenin chloride was released in GP_93_ and then decreased in GP_123_. The SA-GEL microbeads did not release as high a content of oenin chloride as the SA or SA-CHIT microbeads, but there was a constant increase in release during GP, and the highest release was observed at the end of GP_123_. The same behavior as for oenin chloride was observed for peonidin-3-*O*-glucoside chloride. The exception is the OP, where the release of this anthocyanin was only detected from the PRE and from SA microbeads. No anthocyanins were detected during the IP, except oenin chloride and peonidin-3-*O*-glucoside chloride in IP_243_ for the PRE. As expected, the *BI* was successfully determined only after PRE digestion and was 32.7% for oenin chloride and 24.0% for peonidin-3-*O*-glucoside chloride (Figure 7D). Due to the relatively low content, it is possible that the anthocyanins were not successfully encapsulated, but it can also be concluded that they are unstable, since they were not identified during the digestion of PRE.

## 3. Materials and Methods

### 3.1. Chemicals and Reagents

Standards for UHPLC analysis of phenolic compounds (phenolic acids, flavonols, flavan-3-ols, stilbenes, anthocyanins) were obtained from Sigma Aldrich (Saint Louis, MO, USA), Extrasynthese (Genay, France), Acros Organics (Geel, Belgium), and Applihem (Darmstadt, Germany). Standards for UHPLC analysis of sugars were purchased from Acros Organics (Geel, Belgium). UHPLC grade reagents (methanol, acetonitrile, glacial acetic acid) were purchased from J.T. Baker (Arnhem, The Netherlands), Fisher Chemical (Loughborough, United Kingdom), and Macron Fine Chemicals (Gliwice, Poland). Reagents for spectrophotometric determination of total phenolic compounds, total flavonoids, and total proanthocyanidins were purchased from CPA chem (Bogomilovo, Bulgaria), Alfa Aesar GmbH & Co KG (Kandel, Germany), and Acros Organics (Geel, Belgium). Chemicals for encapsulation and simulated digestion (enzymes, bile extract) was obtained from Sigma Aldrich (Saint Louis, MO, USA). Salts for preparations of solutions and buffers were obtained from Acros Organics (Geel, Belgium), Gram Mol (Zagreb, Croatia), and Kemika (Zagreb, Croatia).

### 3.2. Grape Pomace Sample

Cabernet Franc (*Vitis vinifera* L.)-variety grape pomace, left after the vinification process, was collected from a local winery (Erdut Winery, Croatia, 2020 harvest). The grape pomace was air-dried (48 h, 25–27 °C) to reduce the moisture content from 48.60% to 8.41%, and the dried pomace was stored at room temperature. Before use, the grape pomace was ground to a particle size of up to 1 mm using an ultracentrifugal mill (Retsch ZM200, Haan, Germany).

### 3.3. Chemical Composition of Grape Pomace

The chemical composition of grape pomace, including the content of dry matter, free fats, crude proteins, ash, individual sugars, neutral detergent fiber (NDF), acid detergent fiber (ADF), acid detergent lignin (ADL), total organic carbon (TOC, TOC_LE_), and total nitrogen (TN), was determined according to the methods described by Šelo et al. [1]. Table 4 shows the equations according to which the individual components of the chemical composition were calculated. The obtained results were finally calculated on a dry basis of the sample and expressed as mean values of replicates ± standard deviation (SD).

### 3.4. Grape Pomace Extract Preparation

To determine the content of total phenolic compounds (TPC), total flavonoids (TFC), total extractable proanthocyanidins (TPA), and individual phenolic compounds (ICPs), a conventional solid-liquid extraction was performed according to Šelo et al. [1]. Briefly, phenolic compounds were extracted from 1 g of grape pomace with 40 mL of 50% aqueous ethanol solution at 80 °C for 120 min at 200 rpm in a water bath with shaking (Julabo, SW-23, Seelbach, Germany). After extraction, the samples were centrifuged at 11,000× *g* for 10 min (Z 326 K, Hermle Labortechnik GmbH, Germany) and supernatant was concentrated on a rotary evaporator to dryness (Büchi, R-210, Flawil, Switzerland) at 50 °C and 48 mbar. The obtained powder of the phenol-rich grape pomace extract (PRE) was prepared in known concentrations and used for analytics and encapsulation.

### 3.5. Determination of Total Phenolic Compounds

Total phenolic content (TPC), total flavonoid content (TFC), and total extractable proanthocyanidins (TPA) were determined spectrophotometrically according to Martinović et al. [2]. The content of phenolic compounds was expressed as the mean of replicates ± standard deviation (SD).

TPC was determined according to Folin-Ciocalteu method described by Waterhouse [93] with minor modifications. To 3160 µL of distilled water, 40 µL of sample was added and then mixed with 200 µL of Folin-Ciocalteu reagent. After 8 min, 600 µL of sodium carbonate (20%, *w/v*) was added and then incubated at 40 °C for 30 min. At 765 nm, the absorbance of the samples was measured against the blank, and the results were expressed in gallic acid equivalents per weight of PRE (mg_GAE_/100 mg_ext_). The blank was prepared with extraction solvent instead of the sample.

TFC was determined according to the aluminum chloride method described by Marinova et al. [94] with some modifications. To 2 mL of water and 500 µL of the sample, 150 µL of sodium nitrite (5%, *w/v*) was added. After 5 min, 150 µL aluminum chloride (10%, *w/v*) was added and, after exactly 6 min, 1 mL sodium hydroxide (1 M) was added. Then 1.2 mL of distilled water was added and, after shaking the mixture, the absorbance was measured at 510 nm compared to a blank containing water instead of the sample. The results obtained were expressed as (+)-catechin equivalents per weight of PRE (mg_CE_/100 mg_ext_).

TPA was determined according to the slightly modified acid-butanol reaction method described by Škerget et al. [95]. An iron(II)sulphate heptahydrate solution was prepared in an HCl-butanol solution (2:3, *v/v*) and added (5 mL) to 500 µL of the sample. After thorough mixing, the samples were incubated for 15 min in a water bath previously heated to 95 °C. Samples were then cooled under water and absorbance was measured at 540 nm compared to a blank sample prepared using distilled water instead of the sample. The TPA was calculated from the molar weight and molar extinction coefficient of cyanidin, and the results were expressed per weight of PRE (mg/100 mg_ext_).

### 3.6. Determination of Individual Phenolic Compounds

Ultra-high performance liquid chromatography (UHPLC Nexera XR, Shimadzu, Japan) was used to quantify the individual phenolic compounds (phenolic acids, flavan-3-ols, flavonols, stilbenes, and anthocyanins) of PRE and microbeads containing PRE. Separation was performed with a reversed-phase Kinetex^®^ C18 core-shell column (100 × 4.6 mm, 2.6 µm, Phenomenex, Torrance, CA, USA), and a photodiode detector (PDA) was used. Prior to UHPLC analyses (Chromafil Xtra PTFE, Macherey-Nagel GmbH & Co. KG, Dueren, Germany), PRE was dissolved at known concentrations using appropriate solvents and filtered through 0.45-µm membranes. The data obtained were processed using LabSolutions 5.87 software.

Determination of phenolic acids, flavan-3-ols, flavonols, and stilbenes was performed according to Bucić-Kojić et al. [96] using a linear gradient of two phases: A. methanol/acetonitrile (50:50, *v/v*) and B. 1.0% acetic acid in water (*v/v*). With a flow rate of 1 mL/min and at 30 °C, a linear gradient was performed from 5% to 30% B in 25 min, from 30% to 40% B in 10 min, from 40% to 48% B in 5 min, from 48% to 70% B in 10 min, from 70% to 100% B in 5 min, isocratic at 100% B for 5 min, followed by a return to baseline conditions (10 min) and column equilibration (12 min). The injection volume of the sample was 20 µL.

According to method described by Martinović et al. [2], determination of anthocyanins was performed. Briefly, two mobile phases were used: A. water/formic acid/acetonitrile (87:10:3, *v/v/v*) and B. water/formic acid/acetonitrile (40:10:50, *v/v/v*) with gradient program 10 min from 10 to 25% mobile phase B, 5 min from 25 to 31% mobile phase B, 5 min from 31 to 40% mobile phase B, 10 min from 40 to 50% mobile phase B, 10 min from 50 to 100% mobile phase B, and 10 min from 100 to 10% mobile phase B. The injection volume of the sample was 20 μL at a flow rate 0.8 mL/min.

By comparing the UV-Vis spectra and retention times of authentic standards analyzed under the same conditions, individual phenolic compounds were detected and quantified using the calibration curves generated with the external standards. At 503, 513, 517, 523, 526, and 531 nm, anthocyanins, callistephin chloride, kuromanin chloride, peonidin-3-*O*-glucoside chloride, myrtillin chloride, oenin chloride, and petunidin chloride were determined, respectively. Hydroxybenzoic acids at 252–280 nm and hydroxycinnamic acids were determined at 276–277 nm, flavan-3-ols at 273–277 nm, procyanidins at 278 nm, flavonols at 355–372 nm, and stilbenes at 305–323 nm. All experiments were performed in triplicate, and results were expressed as the mean of the replicate ± standard deviation (SD).

### 3.7. Encapsulation by the Ionic Gelation Method

The PRE (1 g) was dissolved in a mixture of 30% aqueous ethanol (20.8 mL) and distilled water (79.2 mL) on a magnetic stirrer for approximately 3 h. To eliminate dry extract particles that remained undissolved, the mixture was centrifuged for 5 min at 15,000× *g*, and 90 mL of clear supernatant was separated for encapsulation while the rest was used for determination of TPC, TFC, TPA, and individual phenolic compounds (samples before digestion).

For ionic gelation, sodium alginate (SA) and combinations of SA with gelatin (GEL) and SA with chitosan (CHIT) were used as coatings. To prepare SA hydrogels, SA was added to the previously prepared supernatant at a concentration of 3% (*w/v*), and the mixture was stirred on a magnetic stirrer for 24 h to allow complete dissolution of the SA, but also to allow sufficient time for binding of active ingredients and coating. Encapsulation was then performed using an encapsulation device (Büchi B-390, Flawil, Switzerland) with a 450 µm diameter nozzle. During encapsulation, the electrode voltage was 750 V and the frequency was 140 Hz, while the pressure was adjusted during the process. Following the “drop-by-drop” principle, the mixture of active ingredient and coating was dropped into 300 mL of freshly prepared crosslinking solution (0.25 M CaCl_2_) and hydrogels were formed. At the end of encapsulation, the hydrogels were allowed to crosslink in CaCl_2_ for 10 min and then filtered through filter paper. After the hydrogels were separated, they were washed twice with 200 mL of distilled water to remove the free calcium ions from the surface of the hydrogels. For the preparation of SA-GEL hydrogels, the same procedure was used, and the concentration of SA was the same (3%, *w/v*) and the concentration of GEL was 5% (*w/v*). To prepare SA-CHIT hydrogels, SA hydrogels were first prepared and then immersed in CHIT after crosslinking in CaCl_2_. CHIT was prepared 24 h before encapsulation by dissolving in 300 mL of 1% acetic acid at a concentration of 1.5% (*w/v*). The produced hydrogels were cooled to −80 °C (SWUF Ultra Low Temperature Smart Freezer, Witeg, Wertheim, Germany) and dried in a freeze-dryer (Freeze-dryer Alpha 2-4 LSCplus, Christ, Osterode am Harz, Germany) at 0.25 mbar for 48 h to achieve better stability of the encapsulated phenolic compounds and to extend the shelf life of the prepared dry microbeads.

After the encapsulation process, the encapsulation efficiency (*EE*) of TPC in hydrogels was calculated according to Equation (15):

(15) $$EE\;\left(\%\right)=\frac{C_{E}-C_{W}}{C_{E}}\times 100$$

where *C_E_* is the TPC measured in the supernatant used for ionic gelation prior to coating(s) addition, and *C_W_* is the TPC found in the remaining mixture of crosslinking solution and wash water. Results are expressed as mean value of replicates ± standard deviation (SD).

### 3.8. Microbead Characterization

#### 3.8.1. Geometric Parameters of Microbeads

Computer image analysis was performed to study the geometric parameters of the microbeads. Exactly 10 microbeads were arranged in a Petri dish so that they did not touch each other, and the Petri dish was placed in a dark chamber on an EPSON V500 Photo Scanner (Epson America Inc., Long Beach, CA, USA). The scanner digitized the samples with a color depth of 24 bits in the sRGB model, with a resolution of 800 dpi in the format TIFF, and after scanning, the images were processed using the ImageJ program (version 1.59g, Wayne Rasband, NIMH, Rockville, MA, USA). The observed parameters were divided into two groups: size and shape parameters. Size parameters included measurements of microbead area, minimum and maximum Feret diameter, and perimeter. The obtained results were converted from pixels to millimeters (mm), taking into account the known values of the scanner resolution in dpi units. The shape of the microbeads was described using the parameters of circularity, roundness and solidity, which provide information on how much the shape of the observed microbeads deviates from a regular circle. All measurements were performed in triplicate with samples taken from different batches. Using the ImageJ User Guide-IJ 1.46r [97], shape parameters were calculated and expressed as the mean of the measurements ± SD.

#### 3.8.2. Scanning Electron Microscopy (SEM)

To investigate microbead morphology, scanning electron microscopy (Hitachi S4700, Hitachi Scientific Ltd., Tokyo, Japan) was used. The samples were coated with a thin layer of gold-palladium film applied using a sputter coater (Bio-Rad SC 502, VG Microtech, Uckfield, UK) and further examined at 10 kV using SEM.

#### 3.8.3. Texture Analysis of Microbeads

Using the TA.XTplus Texture Analyzer (Stable Microsystems Ltd., Surrey, UK) the texture profile of the microbeads was analyzed. Individual microbeads were compressed with a 10 mm diameter aluminum cylinder probe to a compression load of 20% with 0.1 mm/s test speed. The hardness value was determined as the maximum peak height. The hardness of 10 microbeads from each sample was evaluated and expressed as the mean of the measurements ± SD.

#### 3.8.4. X-ray Powder Diffraction (XPRD) 

Using an XPRD system (BRUKER D8 Advance diffractometer, Karlsruhe, Germany), the crystalline structure of the PRE, coatings, and microbeads was investigated. The samples were subjected to Cu Kα radiation (λ = 1.5406 Å) and scanned at 40 kV and 40 mA in the interval of 3–40 2θ with a VÅNTEC-1 detector. The evaluation of the results included smoothing, Kα2-stripping, and background removal, which were performed using DIFFRAC plus EVA software (Karlsruhe, Germany).

#### 3.8.5. Differential Scanning Calorimetry (DSC)

The thermal behavior of the PRE, coatings, and microbeads was investigated using DSC (Mettler Toledo 821e DSC; Mettler Inc., Schwerzenbach, Switzerland). The samples (3–5 mg) were measured at a heating rate of 10 °C/min in the temperature interval of 25–300 °C, under a constant argon flow of 150 mL/min.

### 3.9. In Vitro Simulated Digestion and Bioaccessibility Index (BI)

**Figure 8:**
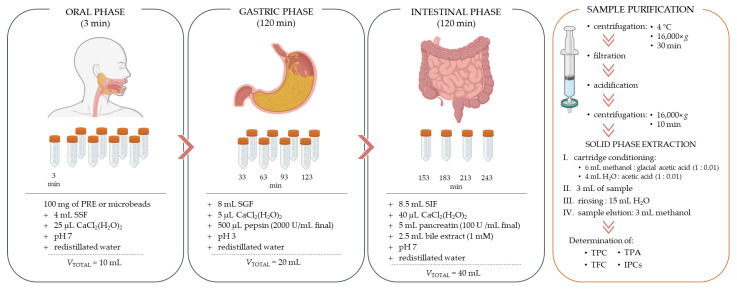
Schematic representation of the implementation of the process of in vitro simulated digestion and purification of the obtained samples.

According to the INFOGEST protocol [98], in vitro simulated digestion of PRE and microbeads containing PRE with different coatings was performed, with modifications. The protocol consists of three steps, i.e., phases: oral (OP), gastric (GP), and intestinal (IP). Figure 8 shows a brief schematic representation of the used protocol. Each of these phases consists of an electrolyte solution that mimics the solutions of the human gastrointestinal tract and whose composition is shown in Table 5.

In vitro simulation of digestion was performed in multiple test tubes, each test tube representing a specific time interval (3, 33, 63, 93, 123, 153, 183, 213, and 243 min). The test tubes were placed on a vertical multi-function rotator (PTR-60, Grant-bio Instruments, UK) located in a thermostat (TC 135 S, Lovibond, Dortmund, Germany) previously heated to 37 °C. Approximately 100 mg of PRE or microbeads containing PRE were weighed into each test tube, and to start the oral phase, 4 mL of SSF solution and 25 µL of CaCl_2_(H_2_O)_2_ were added to each test tube. The pH was adjusted to 7 using 1 M HCl or 1 M NaOH (as needed) and redistilled water was added to a total volume of 10 mL. After 3 min, the test tube representing the OP was removed from the rotator, and 8 mL of SGF solution was added to the remaining test tubes to initiate the gastric phase. Also, 5 µL of CaCl_2_(H_2_O)_2_ was added, the pH of the solution was adjusted to 3, and 500 µL of pepsin was then added. Prior to the gastric phase, pepsin was dissolved in redistilled water and then added so that its activity in the final solution was 2000 U/mL. After addition of the pepsin solution, redistilled water was added so that the total volume of the mixture was 20 mL. At the end of each specific time interval during the GP (33, 63, 93, 123), the test tubes were removed from the rotator. To initiate the intestinal phase, 8.5 mL of SIF solution and 40 µL of CaCl_2_(H_2_O)_2_ were added to the remaining test tubes and the pH was adjusted to 7. Then, 5 mL of pancreatin solution and 2.5 mL of bile extract solution were added, and finally, the volume of the reaction mixture forming the intestinal phase was made up with water to a total volume of 40 mL. The pancreatin solution was prepared prior to the intestinal phase by dissolving pancreatin in SIF solution so that its activity in the final volume was 100 U of trypsin/mL. The bile extract was prepared in the same way, i.e., it was dissolved in the SIF solution before being added to the intestinal phase mixture, so that the concentration of bile extract in the final solution was 1 mM. During the intestinal phase, test tubes were removed from the rotator at specific time intervals (153, 183, 213, and 243 min). Immediately after removing the test tube from rotator at designated time interval, samples were centrifuged at 16,000× *g* and 4 °C for 30 min. Supernatant was removed and filtered through a 0.45 μm membrane (Syringe filters Spheros Nylon, Agilent Technologies, USA). The filtrates contained impurities such as salts, bile extract, and enzyme residues, and were therefore purified by solid phase extraction (SPE) prior to chromatographic analysis. Purification was performed using a modified method according to Kamiloglu et al. [99]. From the previously centrifuged and filtered sample, 4 mL of filtrate was separated and acidified with 80 µL glacial acetic acid (99.5%) to remove any enzyme residues. The acidified samples were centrifuged at 16,000× *g* for 10 min, and the separated clear fraction was then purified. SPE purification cartridges (Superclean LC-18, 100 mg/1 mL, Sigma Aldrich/Supelco) were conditioned with 6 mL of methanol acidified with glacial acetic acid (1:0.01) and 4 mL of distilled water/glacial acetic acid (1:0.01) before use. The centrifuged samples were then passed through conditioned cartridges (3 mL) and washed with redistilled water (15 mL). Samples were then eluted with methanol (3 mL), and the TPC, TFC, TPA, and IPCs were determined (samples during and after digestion).

The bioaccessibility index (BI) was calculated using the following Equation (16):

(16) $$BI,\;\%=\frac{C_{A}}{C_{B}}\times 100$$

where *C_A_* is the content of phenolic compounds in the sample after complete digestion (IP_243_) and *C_B_* is the content of phenolic compounds in PRE before digestion. The phenolic compound content was expressed per 100 mg of the extract.

### 3.10. Statistical Analysis

One-way analysis of variance (ANOVA) was performed using TIBCO Statistica software (TIBCO Software Inc., Palo Alto, CA, USA) to test the significance level of the difference between the arithmetic means of samples representing populations. After ANOVA showed the presence of statistically significant differences between the observed populations, further analysis was performed using a post hoc test, i.e., Duncan’s test for multiple ranges, to determine between which populations there was a significant difference (*p* < 0.05). Samples belonging to the same population were marked with the same letter of the alphabet in the figures or tables.

## 4. Conclusions

Encapsulation of PRE was successfully performed using the ionic gelation technique with the coatings SA, SA-GEL, and SA-CHIT. The results showed that the combined coatings significantly improved the *EE* by 51.90% and 61.64% for SA-GEL and SA-CHIT, respectively, compared with SA. In addition, encapsulation under tested conditions changed the structural properties of the extract from crystalline to amorphous, resulting in better stability and solubility of the active compounds during digestion. Bioaccessibility was also influenced by the coatings tested, and, in general, SA-GEL microbeads provided the highest *BI* of phenolic compounds, followed by SA and SA-CHIT microbeads. It can be supposed, among other things, that the physicochemical properties (size, shape, and texture) of the microbeads produced could be responsible for the outcome of the *BI*. The fate of phenolic compounds in the gastrointestinal tract has not been fully elucidated, but this work provides additional insight and complements previous research. The reason for the lack of knowledge is due to the structural changes that occur during digestion, but also due to the interactions of phenolic compounds with other compounds present in the material undergoing simulated digestion, as well as with constituents present in the administered samples (i.e., food matrix) that may better affect or otherwise interfere with the absorption of a particular phenolic compound and cause lower absorption. Since many parameters can affect the release of phenolic compounds from microbeads, and thus their bioaccessibility, it is necessary to conduct further studies focusing on the effects of coatings on the physical parameters of microbeads and the release and bioaccessibility of phenolic compounds.

## Figures and Tables

**Table 1:** Chemical composition of Cabernet Franc variety grape pomace.

Component	Content (Mean Value ± SD)
Dry matter (%)	91.59 ± 0.02
Crude proteins (%_db_)	8.72 ± 0.26
Free fats (%_db_)	8.54 ± 0.37
Glucose (mg/g_db_)	4.38 ± 0.13
Arabinose (mg/g_db_)	1.50 ± 0.19
Sucrose (mg/g_db_)	9.87 ± 0.17
Fructose (mg/g_db_)	4.94 ± 0.04
Ash (%_db_)	7.16 ± 0.15
NDF (%_db_)	47.24 ± 0.56
ADF (%_db_)	37.60 ± 0.36
ADL (lignin) (%_db_)	23.77 ± 0.26
Hemicellulose (%_db_)	9.64 ± 0.55
Cellulose (%_db_)	13.82 ± 0.62
TOC (mg/g_db_)	59.22 ± 0.49
TOC_LE_ (mg/g_db_)	53.32 ± 1.13
TN (mg/g_db_)	0.96 ± 0.03

db—dry basis, NDF—neutral detergent fibers, ADF—acid detergent fibers, ADL—acid detergent lignin, TOC—total organic carbon in crude grape pomace, TOC_LE_—total organic carbon in liquid grape pomace extract, TN—total nitrogen. All data are expressed as mean value of replication (*n* = 3) ± SD.

**Table 2:** Values for size parameters, shape parameters, and texture parameters of microbeads containing phenol-rich grape pomace extract prepared using sodium alginate (SA), SA and gelatin (SA-GEL), and SA and chitosan (SA-CHIT) as coatings.

Sample	Size Parameters				Shape Parameters			Texture
	Area (mm^2^)	Perimeter (mm)	Feret_MAX_ (mm)	Feret_MIN_ (mm)	Circularity (-)	Roundness (-)	Solidity (-)	Hardness (N)
SA	6.62 ± 1.72 a	10.25 ± 1.26 a	3.39 ± 0.38 a	2.68 ± 0.36 a	0.79 ± 0.06 b	0.80 ± 0.08 a	0.96 ± 0.02 b	1.36 ± 0.66 b
SA-GEL	11.18 ± 0.96 c	13.79 ± 0.86 c	4.24 ± 0.27 b	3.53 ± 0.18 c	0.74 ± 0.07 a	0.86 ± 0.08 b	0.97 ± 0.01 c	0.49 ± 0.20 a
SA-CHIT	7.63 ± 2.71 b	11.37 ± 2.37 b	3.51 ± 0.64 a	2.90 ± 0.49 b	0.72 ± 0.07 a	0.86 ± 0.06 b	0.94 ± 0.02 a	0.56 ± 0.26 a

All data are expressed as mean value of replication ± SD. Different letters (a, b, c) represent statistically significant differences between the coatings used for each size, shape, or texture parameter (ANOVA, post-hoc Duncan test at *p* < 0.05).

**Table 3:** Content of individual phenolic compounds of phenol-rich grape pomace extract (PRE), sodium alginate microbeads (SA), sodium alginate whit gelatin microbeads (SA-GEL), and sodium alginate whit chitosan microbeads (SA-CHIT) before digestion (BD) and during three phases of in vitro simulated digestion.

Component	Sample	Before Digestion	Oral Phase	Gastric Phase				Intestinal Phase			
		BD	OP_3_	GP_33_	GP_63_	GP_93_	GP_123_	IP_153_	IP_183_	IP_213_	IP_243_
***Phenolic acids* (μg/100 mg_ext_)**											
Gallic acid	PRE	130.00 ± 10.98	2.09 ± 0.06	-	-	-	1.19 ±0.17	-	-	-	141.38 ± 6.37
	SA		4.33 ± 1.49	10.78 ± 0.76	10.77 ± 0.62	9.66 ± 0.27	13.40 ± 0.27	260.87 ± 48.60	146.83 ± 8.58	229.33 ± 7.36	170.34 ± 2.39
	SA-GEL		2.72 ± 0.80	2.24 ± 0.60	12.31 ± 0.84	nd	8.52 ± 0.46	906.35 ± 168.35	455.08 ± 7.73	459.47 ± 15.62	431.10 ± 25.41
	SA-CHIT		1.00 ± 0.07	3.09 ± 0.17	12.44 ± 0.07	8.67 ± 0.30	8.94 ± 0.40	238.41 ± 5.11	181.35 ± 6.75	115.47 ± 7.02	158.57 ± 14.96
3,4-Dihydroxy-benzoic acid	PRE	24.09 ± 1.97	nd	-	-	-	nd	-	-	-	17.01 ± 0.08
	SA		nd	nd	nd	nd	nd	99.46 ± 2.40	81.70 ± 1.62	80.04 ± 1.63	55.94 ± 0.07
	SA-GEL		nd	nd	nd	nd	nd	100.70 ± 4.98	76.46 ± 1.99	84.24 ± 0.61	75.93 ± 1.07
	SA-CHIT		nd	nd	nd	nd	nd	129.42 ± 37.35	74.47 ± 0.55	40.28 ± 2.58	74.01 ± 0.20
Syringic acid	PRE	114.36 ± 3.02	6.92 ± 0.78	-	-	-	nd	-	-	-	nd
	SA		5.98 ± 0.26	7.79 ± 0.51	4.46 ± 0.48	2.98 ± 0.00	3.79 ± 0.14	nd	nd	nd	nd
	SA-GEL		5.97 ± 0.56	5.97 ± 0.13	4.53 ± 0.07	6.04 ± 0.61	5.68 ± 0.21	nd	nd	nd	nd
	SA-CHIT		3.54 ± 0.12	3.30 ± 0.10	3.11 ± 0.85	2.58 ± 0.54	1.48 ± 0.34	nd	nd	nd	nd
Vanillic acid	PRE	14.83 ± 0.74	nd	-	-	-	nd	-	-	-	4.39 ± 0.00
	SA		nd	nd	nd	nd	nd	3.87 ± 0.00	3.39 ± 0.20	3.56 ± 0.41	3.34 ± 0.21
	SA-GEL		nd	nd	nd	nd	nd	nd	nd	nd	nd
	SA-CHIT		nd	nd	nd	nd	nd	nd	nd	nd	nd
Ellagic acid	PRE	81.33 ± 1.23	nd	-	-	-	nd	-	-	-	1.28 ± 0.00
	SA		1.72 ± 0.17	1.50 ± 1.03	1.21 ± 0.07	11.08 ± 0.24	10.63 ± 0.07	11.52 ± 0.14	8.84 ± 0.34	7.03 ± 0.27	7.59 ± 0.48
	SA-GEL		nd	nd	4.33 ± 0.07	6.19 ± 0.61	7.74 ± 0.38	13.33 ± 0.94	12.48 ± 0.34	12.46 ± 1.29	11.52 ± 0.40
	SA-CHIT		nd	1.59 ± 0.34	8.10 ± 0.00	10.62 ± 0.68	12.08 ± 0.77	13.30 ± 1.10	10.00 ± 0.41	9.32 ± 0.14	8.55 ± 0.88
*p*-Hydroxybenzoic acid	PRE	1.00 ± 0.04	nd	-	-	-	nd	-	-	-	nd
	SA		nd	nd	nd	nd	nd	nd	nd	nd	nd
	SA-GEL		nd	nd	nd	nd	nd	nd	nd	nd	nd
	SA-CHIT		nd	nd	nd	nd	nd	nd	nd	nd	nd
*o*-Coumaric acid	PRE	19.48 ± 0.58	nd	-	-	-	nd	-	-	-	9.37 ± 0.06
	SA		nd	3.52 ± 1.00	5.19 ± 0.34	29.70 ± 0.14	28.23 ± 0.31	82.37 ± 5.20	94.99 ± 1.49	87.12 ± 1.02	99.36 ± 4.17
	SA-GEL		nd	nd	10.69 ± 0.61	13.83 ± 0.71	20.34 ± 0.48	66.87 ± 6.26	75.19 ± 3.91	81.98 ± 7.21	87.50 ± 0.73
	SA-CHIT		nd	4.46 ± 0.31	21.11 ± 1.02	30.61 ± 1.83	36.68 ± 2.22	90.29 ± 6.32	104.17 ± 0.55	105.93 ± 2.99	123.03 ± 7.91
*p*-Coumaric acid	PRE	1.44 ± 0.04	0.43 ± 0.06	-	-	-	0.44 ± 0.00	-	-	-	3.93 ± 0.70
	SA		nd	nd	nd	nd	nd	nd	nd	nd	nd
	SA-GEL		nd	nd	nd	nd	nd	nd	nd	nd	nd
	SA-CHIT		nd	nd	nd	nd	nd	nd	nd	nd	nd
Caffeic acid	PRE	1.04 ± 0.02	nd	-	-	-	nd	-	-	-	nd
	SA		nd	nd	nd	nd	nd	nd	nd	nd	nd
	SA-GEL		nd	nd	nd	nd	nd	nd	nd	nd	nd
	SA-CHIT		nd	nd	nd	nd	nd	nd	nd	nd	nd
Ferulic acid	PRE	2.52 ± 0.25	nd	-	-	-	nd	-	-	-	nd
	SA		nd	nd	nd	nd	nd	nd	nd	nd	nd
	SA-GEL		nd	nd	nd	nd	nd	nd	nd	nd	nd
	SA-CHIT		nd	nd	nd	nd	nd	nd	nd	nd	nd
***Stilbenes* (μg/100 mg_ext_)**											
Resveratrol	PRE	8.82 ± 0.04	nd	-	-	-	nd	-	-	-	nd
	SA		nd	nd	nd	nd	nd	nd	nd	nd	nd
	SA-GEL		nd	nd	nd	nd	nd	nd	nd	nd	nd
	SA-CHIT		nd	nd	nd	nd	nd	nd	nd	nd	nd
ε-viniferin	PRE	13.72 ± 0.06	nd	-	-	-	nd	-	-	-	nd
	SA		nd	nd	nd	nd	nd	nd	nd	nd	nd
	SA-GEL		nd	nd	nd	nd	nd	nd	nd	nd	nd
	SA-CHIT		nd	nd	nd	nd	nd	nd	nd	nd	nd
***Flavan-3-ols* (μg/100 mg_ext_)**											
Epicatechin	PRE	547.27 ± 25.23	343.19 ± 7.06	-	-	-	324.16 ± 4.16	-	-	-	nd
	SA		103.46 ± 3.19	135.12 ± 11.02	96.92 ± 5.59	140.82 ± 2.79	157.62 ± 9.34	573.86 ± 13.62	624.57 ± 6.62	685.58 ± 11.71	637.35 ± 3.56
	SA-GEL		64.03 ± 1.00	84.71 ± 1.27	95.96 ± 3.64	126.00 ± 3.67	133.18 ± 1.68	561.73 ± 0.74	634.47 ± 20.60	581.18 ± 22.59	550.13 ± 18.30
	SA-CHIT		72.78 ± 14.36	84.77 ± 2.28	117.12 ± 12.92	127.29 ± 3.08	104.42 ± 10.91	541.26 ± 38.72	742.62 ± 2.33	619.73 ± 6.12	790.51 ± 29.93
Catechin	PRE	527.59 ± 28.62	100.95 ± 2.01	-	-	-	139.84 ± 2.90	-	-	-	nd
	SA		97.24 ± 0.12	108.54 ± 15.03	59.50 ± 7.27	61.06 ± 2.89	89.93 ± 5.11	nd	nd	nd	nd
	SA-GEL		43.37 ± 2.35	37.47 ± 0.57	32.03 ± 1.29	49.26 ± 0.61	71.02 ± 1.24	nd	nd	nd	nd
	SA-CHIT		72.07 ± 12.00	61.44 ± 3.31	54.76 ± 4.43	67.08 ± 0.03	44.18 ± 1.11	nd	nd	nd	nd
Epicatechin gallate	PRE	32.46 ± 1.65	nd	-	-	-	nd	-	-	-	32.06 ± 0.06
	SA		1.78 ± 0.29	17.79 ± 0.17	28.90 ± 2.74	252.14 ± 1.70	253.28 ±.9.30	205.06 ± 8.69	152.85 ± 0.47	133.54 ± 7.29	112.17 ± 6.02
	SA-GEL		nd	10.69 ± 0.17	63.98 ± 2.08	82.26 ± 6.63	118.13 ± 1.06	100.66 ± 5.05	93.06 ± 1.03	69.38 ± 1.63	66.02 ± 2.94
	SA-CHIT		nd	34.80 ± 0.14	183.86 ± 21.13	278.40 ± 10.69	300.12 ± 3.77	261.80 ± 22.31	224.08 ± 0.14	183.36 ± 6.59	155.47 ± 6.08
Gallocatechin gallate	PRE	127.23 ± 9.54	nd	-	-	-	nd	-	-	-	190.83 ± 6.03
	SA		nd	nd	nd	nd	nd	545.98 ± 29.50	535.41 ± 8.65	518.14 ± 3.47	482.73 ± 10.39
	SA-GEL		nd	nd	nd	nd	nd	391.35 ± 0.47	417.09 ± 8.38	430.16 ± 15.58	423.44 ± 1.14
	SA-CHIT		89.18 ± 3.50	169.71 ± 2.69	318.62 ± 32.48	320.13 ± 12.21	212.59 ± 2.05	516.89 ± 5.77	511.21 ± 5.01	489.47 ± 1.63	510.90 ± 13.45
Procyanidin B1	PRE	317.42 ± 2.59	60.20 ± 0.10	-	-	-	72.36 ± 7.13	-	-	-	nd
	SA		30.94 ± 1.54	nd	nd	nd	nd	nd	nd	nd	nd
	SA-GEL		nd	nd	nd	nd	nd	nd	nd	nd	nd
	SA-CHIT		nd	nd	nd	nd	nd	nd	nd	nd	nd
Procyanidin B2	PRE	126.51 ± 23.32	111.71 ± 5.15	-	-	-	137.25 ± 3.75	-	-	-	nd
	SA		34.94 ± 2.09	65.53 ± 0.62	33.29 ± 4.70	73.27 ± 1.46	146.07 ± 5.77	41.72 ± 3.70	nd	nd	nd
	SA-GEL		nd	nd	12.88 ± 0.44	16.63 ± 0.64	27.06 ± 2.57	nd	nd	nd	nd
	SA-CHIT		39.02 ± 8.96	33.09 ± 1.19	77.44 ± 7.33	126.02 ± 4.94	108.64 ± 4.75	nd	nd	nd	nd
***Flavonols* (μg/100 mg_ext_)**											
Quercetin	PRE	146.04 ± 3.93	19.62 ± 0.67	-	-	-	15.89 ± 0.38	-	-	-	nd
	SA		nd	nd	nd	nd	nd	nd	nd	nd	nd
	SA-GEL		nd	nd	nd	nd	nd	nd	nd	nd	nd
	SA-CHIT		nd	nd	nd	nd	nd	nd	nd	nd	nd
Rutin	PRE	65.08 ± 5.10	46.81 ± 0.68	-	-	-	64.19 ± 0.00	-	-	-	18.86 ± 1.80
	SA		nd	nd	nd	nd	nd	nd	nd	nd	nd
	SA-GEL		nd	nd	nd	nd	nd	nd	nd	nd	nd
	SA-CHIT		nd	nd	nd	nd	nd	nd	nd	nd	nd
Kaempferol	PRE	10.40 ± 1.00	nd	-	-	-	nd	-	-	-	nd
	SA		nd	nd	nd	nd	nd	nd	nd	nd	nd
	SA-GEL		nd	nd	nd	nd	nd	nd	nd	nd	nd
	SA-CHIT		nd	nd	nd	nd	nd	nd	nd	nd	nd
***Anthocyanins* (μg/100 mg_ext_)**											
Oenin chloride	PRE	794.37 ± 21.84	511.54 ± 1.17	-	-	-	733.77 ± 1.11	-	-	-	259.78 ± 23.24
	SA		20.00 ± 3.19	102.91 ± 0.07	107.27 ± 0.07	139.91 ± 0.54	123.64 ± 1.92	nd	nd	nd	nd
	SA-GEL		1.63 ± 0.07	70.75 ± 0.30	79.32 ± 0.68	86.93 ± 0.10	88.30 ± 3.71	nd	nd	nd	nd
	SA-CHIT		nd	109.96 ± 1.36	121.79 ± 0.17	65.26 ± 0.17	90.66 ± 1.75	nd	nd	nd	nd
Myrtillin chloride	PRE	35.15 ± 0.00	7.98 ± 0.30	-	-	-	6.73 ± 0.01	-	-	-	nd
	SA		nd	nd	nd	nd	nd	nd	nd	nd	nd
	SA-GEL		nd	nd	nd	nd	nd	nd	nd	nd	nd
	SA-CHIT		nd	nd	nd	nd	nd	nd	nd	nd	nd
Petunidin chloride	PRE	7.77 ± 0.01	3.11 ± 0.17	-	-	-	3.62 ± 0.22	-	-	-	nd
	SA		nd	nd	nd	nd	nd	nd	nd	nd	nd
	SA-GEL		nd	nd	nd	nd	nd	nd	nd	nd	nd
	SA-CHIT		nd	nd	nd	nd	nd	nd	nd	nd	nd
Peonidin-3-*O*-glucoside chloride	PRE	77.75 ± 7.28	45.42 ± 0.90	-	-	-	61.59 ± 1.32	-	-	-	18.64 ± 2.23
	SA		1.16 ± 0.67	8.28 ± 0.65	10.04 ± 0.07	11.51 ± 0.31	9.61 ± 0.27	nd	nd	nd	nd
	SA-GEL		nd	5.21 ± 0.34	6.00 ± 0.03	5.88 ± 0.71	6.00 ± 0.38	nd	nd	nd	nd
	SA-CHIT		nd	6.58 ± 0.65	7.21 ± 0.10	3.04 ± 0.10	6.38 ± 0.54	nd	nd	nd	nd
Kuromanin chloride	PRE	4.88 ± 0.03	nd	-	-	-	nd	-	-	-	nd
	SA		nd	nd	nd	nd	nd	nd	nd	nd	nd
	SA-GEL		nd	nd	nd	nd	nd	nd	nd	nd	nd
	SA-CHIT		nd	nd	nd	nd	nd	nd	nd	nd	nd
Callistephin chloride	PRE	2.27 ± 0.03	nd	-	-	-	nd	-	-	-	nd
	SA		nd	nd	nd	nd	nd	nd	nd	nd	nd
	SA-GEL		nd	nd	nd	nd	nd	nd	nd	nd	nd
	SA-CHIT		nd	nd	nd	nd	nd	nd	nd	nd	nd

GP—gastric phase, IP—intestinal phase, OP—oral phase, nd—not detected, “-”—not determined. Index numbers associated with abbreviations indicate the time interval when a certain sample was taken (i.e., GP_33_—33rd minute of the gastric phase). For the PRE, only the endpoints of the oral, gastric, and intestinal phases are shown (OP_3_, GP_123_, IP_243_). Phenolic contents are expressed as mean value (μg /100 mg_ext_) ± SD.

**Table 4:** Equations for calculating the value of individual components of the grape pomace chemical composition.

Analysis	Equation		Marks
Dry matter	$w\;\left(\%\right)=\frac{m_{2}}{m_{1}}\times 100$	(1)	*w*—dry matter content (%), *m*_1_—sample mass before drying (g), *m*_2_—sample mass after drying (g)
Free fats	$Free\;fats\;\left(\%\right)=\frac{m_{E}}{m_{S}}\times 100$	(2)	*m*_E_—mass of the extract containing free fats obtained after Soxhlet extraction (g), *m*_S_—sample mass used for Soxhlet extraction (g)
Crude proteins	$N\;\left(\%\right)=\frac{\left(a-b\right)\times f\times 0.14}{c\times 10}$	(3)	*N*—nitrogen content (%), *a*—volume of 0.01 M sodium hydroxide used for titration of the blank sample (mL), *b*—volume of 0.01 M sodium hydroxide used for titration of the tested sample (mL), *f*—factor of 0.01 M sodium hydroxide used (*f* = 1), *c*—amount of sample (g)
	$Crude\;proteins\;\left(\%\right)=N\times 6.25$	(4)	
Ash	$Ash\;\left(\%\right)=\frac{m_{3}-m_{1}}{m_{2}-m_{1}}\times 100$	(5)	*m*_1_—empty crucible mass (g), *m*_2_—mass of crucible with sample (g), *m_3_*—mass of crucible after combustion (g)
Individual sugars	$P=210,520.91\times C_{G}-18.08$	(6)	*P*—area under the peak, *C*—mass concentration of individual sugar (mg/L), G—glucose, A—arabinose, S—sucrose, F—fructose
	$P=218,160.63\times C_{A}+31,070.06$	(7)	
	$P=100,128.34\times C_{S}+11.10$	(8)	
	$P=156,272.11\times C_{F}+28.50$	(9)	
Fibers	$NDF,\;ADF\;\left(\%\right)=\frac{m_{2}}{m_{1}}\times 100$	(10)	*NDF*—neutral detergent fibers (%), *ADF*—acid detergent fibers (%)*, m*_1_—residue after drying (g), *m*_S_—sample mass (g), *ADL* (lignin)—acid detergent lignin (%), *m*_1_—residue after drying (g), *m*_2_—residue after combustion (g), *m*_S_—sample mass (g)
	$ADL\;\left(\%\right)=\frac{m_{1}-m_{2}}{m_{S}}\times 100$	(11)	
	$Hemicellulose\;\left(\%\right)=NDF-ADF$	(12)	
	$Cellulose\;\left(\%\right)=ADF-ADL$	(13)	
Total organic carbon	$TOC\;\left(\%\right)=TC-IC$	(14)	*TOC*—total organic carbon (%), *TC*—total carbon (%), *IC*—inorganic carbon (%)

**Table 5:** The composition of the solutions for in vitro simulated digestion with the corresponding concentrations of the components in the final solution.

Constituents	Simulated Salivary Fluid	Simulated Gastric Fluid	Simulated Intestinal Fluid
	(SSF)	(SGF)	(SIF)
KCl (mM)	15.1	6.9	6.8
KH_2_PO_4_ (mM)	3.7	0.9	0.8
NaHCO_3_ (mM)	13.6	25	85
NaCl (mM)	-	47.2	38.4
MgCl_2_(H_2_O)_6_ (mM)	0.15	0.1	0.33
(NH_4_)_2_CO_3_ (mM)	0.06	0.5	-
CaCl_2_(H_2_O)_2_ (mM)	1.5	0.15	0.6

## Data Availability

Not applicable.
